# Assessing the Performance of Chat Generative Pretrained Transformer (ChatGPT) in Answering Andrology-Related Questions

**DOI:** 10.5152/tud.2023.23171

**Published:** 2023-11-01

**Authors:** Ufuk Caglar, Oguzhan Yildiz, M. Fırat Ozervarli, Resat Aydin, Omer Sarilar, Faruk Ozgor, Mazhar Ortac

**Affiliations:** 1 Department of Urology, Haseki Training and Research Hospital, Istanbul, Turkey; 2 Department of Urology, Istanbul University, Istanbul School of Medicine, Istanbul, Turkey

**Keywords:** Andrology, artificial intelligence, information sources

## Abstract

**Objective:**

The internet and social media have become primary sources of health information, with men frequently turning to these platforms before seeking professional help. Chat generative pretrained transformer (ChatGPT), an artificial intelligence model developed by OpenAI, has gained popularity as a natural language processing program. The present study evaluated the accuracy and reproducibility of ChatGPT's responses to andrology-related questions.

**Methods:**

The study analyzed frequently asked andrology questions from health forums, hospital websites, and social media platforms like YouTube and Instagram. Questions were categorized into topics like male hypogonadism, erectile dysfunction, etc. The European Association of Urology (EAU) guideline recommendations were also included. These questions were input into ChatGPT, and responses were evaluated by 3 experienced urologists who scored them on a scale of 1 to 4.

**Results:**

Out of 136 evaluated questions, 108 met the criteria. Of these, 87.9% received correct and adequate answers, 9.3% were correct but insufficient, and 3 responses contained both correct and incorrect information. No question was answered completely wrong. The highest correct answer rates were for disorders of ejaculation, penile curvature, and male hypogonadism. The EAU guideline-based questions achieved a correctness rate of 86.3%. The reproducibility of the answers was over 90%.

**Conclusion:**

The study found that ChatGPT provided accurate and reliable answers to over 80% of andrology-related questions. While limitations exist, such as potential outdated data and inability to understand emotional aspects, ChatGPT's potential in the health-care sector is promising. Collaborating with health-care professionals during artificial intelligence model development could enhance its reliability.

**Main Points**

- The present study showed that chat generative pretrained transformer gave highly accurate answers to frequently asked questions by patients about andrology.
- In addition, the AI model performed similarly satisfactorily in questions of evidence-based medicine.
- Artificial intelligence technology can take an important place in the health sector in the future.

## Introduction

Men may be reluctant to discuss their health problems, concerns, and fears with health professionals. This hesitation is especially evident in issues related to men’s health.^1^ The internet and social media are frequently used by patients as sources of health information. Men especially turn to the internet for health information before professionals.^2^ Social media (Youtube, Facebook, Instagram, Twitter, etc.) and artificial intelligence (AI) applications that have become popular in recent years are the first sources that come to mind in this regard.

Chat generative pretrained transformer (ChatGPT), a natural language processing program developed by OpenAI, is one of the most frequently used AI programs.^3^ The increasing popularity of ChatGPT has paved the way for research on the effectiveness of its application in the field of health. Bonetti et al^4^ showed that ChatGPT achieved a high success rate of 87% accuracy in the Italy Residency Admission National Exam. In another study, Deiana et al^5^ demonstrated that ChatGPT provided a high rate of correct answers to questions related to public health.^5^

Although some studies have shown the effectiveness of ChatGPT on different medical topics, its adequacy for questions related to andrology and men’s health has not been previously evaluated. In this study, we aimed to evaluate the adequacy of ChatGPT responses to questions related to andrology.

## Material and Methods

Frequently asked questions about andrology by patients on health forums, hospital websites, and social media (YouTube and Instagram) were analyzed. In selecting the sources for determining the questions, we aimed for a diverse and representative sample of platforms where patients typically seek information related to andrology. Only questions in English were included in the study. Questions were categorized by topic (male hypogonadism, erectile dysfunction, disorders of ejaculation, low sexual desire and male hypoactive sexual desire disorder, penile curvature, penile size abnormalities and dysmorphophobia, priapism, and male infertility). In addition, the recommendation tables of the Sexual and Reproductive Health section of the 2023 European Association of Urology (EAU) guidelines were analyzed.^6^ Those with a strong recommendation level were translated into a question form. All questions were asked of the ChatGPT 3.5 August version in English. The responses generated by AI were noted. All questions were asked again twice at different times during the day to evaluate the reproducibility of the answers.

Responses were evaluated by 3 urologists experienced in andrology. The reviewers scored the responses on a scale of 1-4. The reviewers scored the responses in comparison to how they would have answered if they had been asked this question by a patient.

4: Correct and adequate answer (no further information to add)
3: Correct answer but insufficient (more detailed explanation required)
2: Accurate and misleading information together
1: Wrong or irrelevant answer

The median score was recorded for questions in which not all reviewers gave the same score. Repeatability was defined as the consistency of the answer given to the same question at different times. Responses generated at different times were considered reproducible if they received the same score. The median score was noted for questions that received different answers when repeated. Questions that varied from person to person were not included in the study (e.g., I am 35 years old; can I have children?). Other exclusion criteria were questions with similar meanings, questions that did not conform to language rules, and non-medical questions. Since no patient data were used in the study, ethics committee approval was not required.

### Statistical analysis

Excel version 16.0 (Microsoft Corp.; Washington, USA) was used for the statistical analyses. The scores of the responses were expressed as n (%). Reproducibility of responses was expressed as %.

## Results

**Figure 1.**
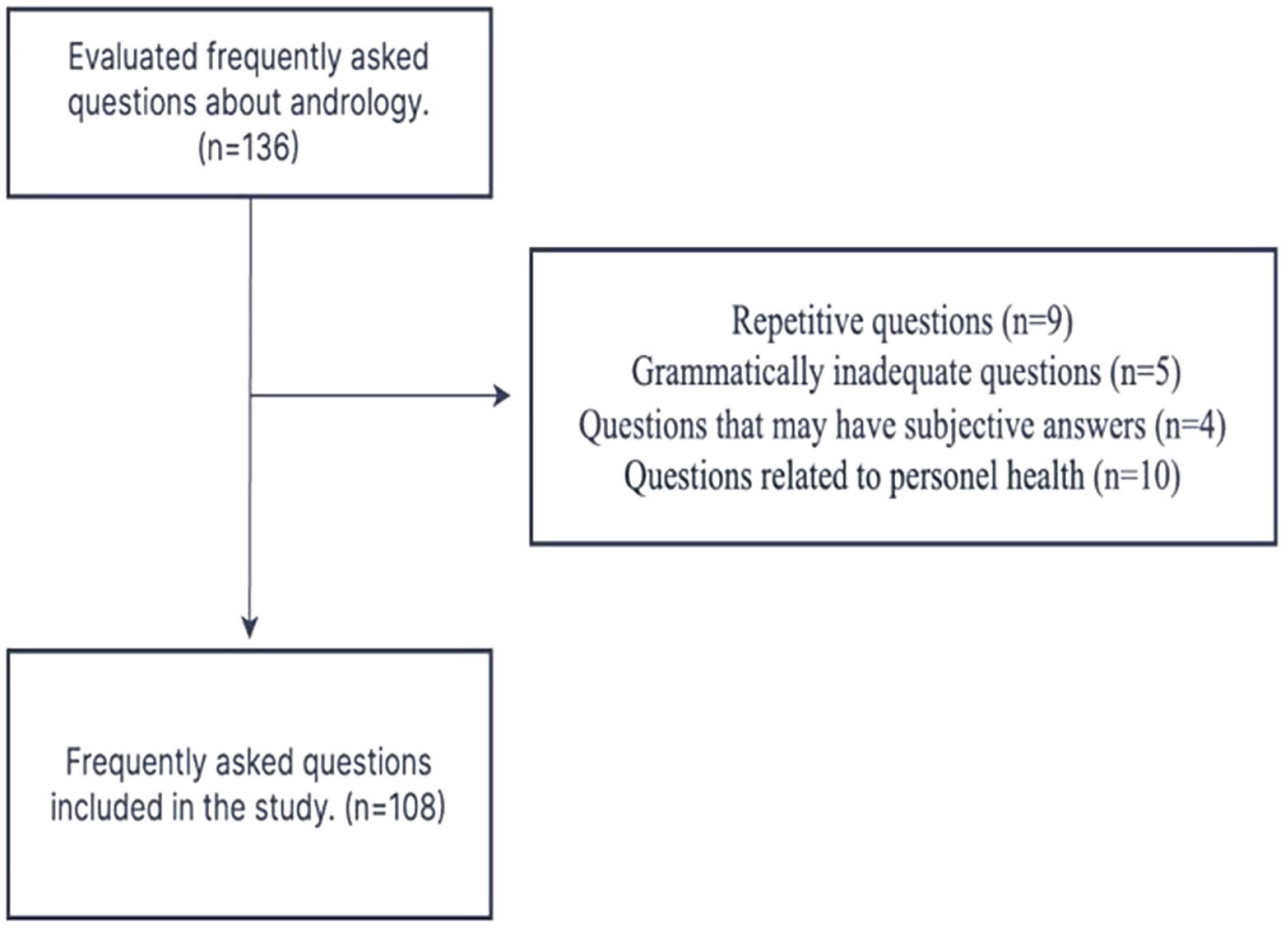
Flowchart of questions included in the study.

The flowchart for the questions included in the study is shown in Figure 1. Of the 136 questions evaluated, 28 did not meet the inclusion criteria. Answers to 108 questions were included in the study (Supplementary Table 1). Ninety-five (87.9%) of the questions were answered correctly and adequately. Ten answers (9.3%) were correct but inadequate, and 3 answers contained both correct and incorrect information. No question was answered incorrectly. The topics of the questions with the highest rate of correct answers were disorders of ejaculation (92.9%), penile curvature (92.9%), and male hypogonadism (91.7%). Erectile dysfunction, penile size abnormalities, and male infertility each had 1 correct answer and 1 incorrect answer. Eighty questions were prepared according to EAU guideline recommendations (Supplementary Table 2). Of the questions, 86.3 were completely correct. Eight questions (10%) received 3 points, and 3 questions (3.8%) received 2 points. All questions regarding ejaculation disorders were answered correctly. The lowest completely correct response rate was for questions about male hypogonadism. Similar to the frequently asked questions, there were no completely wrong answers in the guideline recommendations (Table 1).

**Figure 2.**
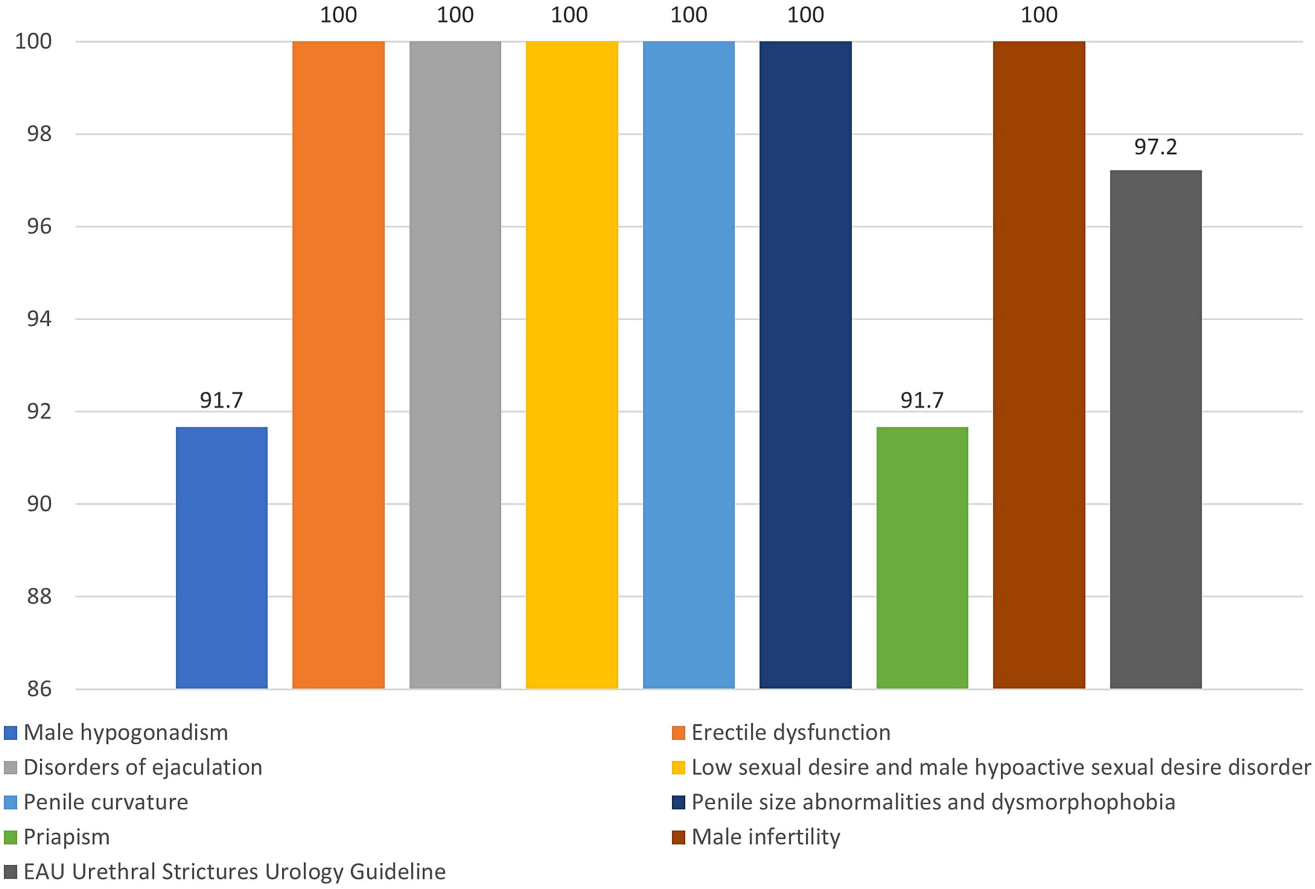
Similarity rates of answers to questions. EAU, European Association of Urology.

The reproducibility rates of the answers to the questions are shown in Figure 2. The AI model produced similar answers for all questions related to erectile dysfunction, disorders of ejaculation, low sexual desire, penile curvature, penile size abnormalities, and male infertility. The similarity rates for the answers to questions about male hypogonadism and priapism were 91.7%. The similarity rate for the questions prepared according to the EAU guideline recommendations was 97.2%.

## Discussion

Artificial intelligence models stand out with their increasing use in many areas of life. The utilization of AI within the realm of health-care has emerged as a notably significant topic in recent times.^7^ This study evaluated the accuracy and reliability of ChatGPT in responding to questions related to andrology. Some research aimed at evaluating the feasibility of utilizing AI-driven platforms to address patients’ questions and concerns has been conducted in various medical fields. Lee et al^8^ revealed that ChatGPT provided satisfactory responses to patient questions about colonoscopy. Samaan et al^9^ showed that the model gave 86.8% correct answers to questions related to bariatric surgery. Our study revealed the impressive performance of ChatGPT in questions related to andrology.

Social media has taken its place as one of the most important information sources for people in the field of health, as in many different fields. Zaila et al^10^ evaluated the characteristics of YouTube videos related to men’s health. They found that, in addition to quality videos, there are many videos with advertising purposes and biases. Dubin et al^11^ found that only about 10% of TikTok and Instagram content related to men’s health was uploaded by health professionals. They stated that the remaining videos contained a high rate of misinformation.^11^ Our study showed that the answers generated by ChatGPT were correct at a rate of 87.9%. The ability of AI software to access the literature and its self-improving structure are important reasons for the high rate of correct answers. In addition, the fact that the information provided is prepared without the concern of advertisements minimizes the bias rate of the answers.

Our results showed that ChatGPT gave highly accurate answers to questions based on EAU guideline recommendations, as well as questions frequently asked by patients. Since the guidelines contain comprehensive and detailed medical information, the success of AI in this regard is remarkable. Ali et al^12^ tested ChatGPT with neurosurgery written board examinations questions and showed that the application obtained enough points to pass the exam.^12^ Panthier et al^13^ applied the ophthalmology board exam to ChatGPT in the French language and showed that AI was 91% successful. ChatGPT can provide accurate answers to challenging medical questions thanks to its access to many scientific articles and book contents. Its ability to generate answers in many commonly spoken languages also allows it to appeal to a wide population. 

An online information source should be easily understandable and easily accessible, apart from its high accuracy rate. ChatGPT’s answers to the questions are in colloquial language and easy to understand.^14^ Understanding the information accessed from search engines can be tiring for patients. In addition, the reproducibility of the answers generated by AI is another important issue. Yeo et al^15^ showed that ChatGPT’s answers to frequently asked questions about cirrhosis and hepatocellular carcinoma were reproducible at around 90%.^15^ Our results showed that ChatGPT’s answers to questions related to andrology were highly reproducible.

Although ChatGPT has a high rate of correct answers to questions related to andrology, it has some inadequacies. The application only includes data from 2021 and before. Since the literature on andrology is constantly renewed, ChatGPT may be insufficient to access up-to-date information. In addition, we do not know the details of the database used by the application. The model does not have personal medical experience. Therefore, it may be difficult for ChatGPT to understand important aspects, such as patient experiences, emotional states, or subjective approaches. Terms, procedures, and details in the medical field are extremely complex. It may be difficult to understand and correctly use this specialized terminology and details. Finally, medical topics often involve personal and sensitive information. A model such as ChatGPT may have limitations in understanding ethical and confidentiality rules and producing sensitive responses.

Our study has some limitations. First, the questions selected for analysis may not fully represent the scope of the research encountered in clinical practice. Categorization of questions according to specific andrological topics may cause bias in the selection process. The categories selected for analysis may not cover the full range of andrological concerns expressed by patients. While responses from the ChatGPT were evaluated by experienced urologists, human judgment was inherently subjective. While efforts have been made to minimize this subjectivity by having more than 1 urologist evaluate the responses, differences in interpretation may still affect the scoring process. Factors such as updates to the model or changes in training data could potentially affect response consistency. Additionally, the questions were evaluated only in English; similar quality responses may not be received to questions asked in other languages. We did not assess intraobserver and interobserver variability among reviewers. Finally, the assessment of response quality was based on a 4-point scoring system, which, despite its structure, can still be influenced by the examiner’s subjectivity.

The results of the current study showed for the first time that ChatGPT provided adequate answers to over 80% of the andrology FAQs. Although it has limitations, it can be predicted that it will take an important place in the health sector in the future, as it is a constantly developing platform. Getting support from health-care professionals in the development of AI models can increase the reliability of the model.

## Figures and Tables

**Table 1.** Grade of Responses By the Chat Generative Pretrained Transformer to the Questions Related to Andrology

	4 Point	3 Point	2 Point	1 Point
Frequently asked questions (n=108)	95 (87.9%)	10 (9.3%)	3 (2.8%)	–
Male hypogonadism (n=12)	11 (91.7%)	1 (8.3%)	–	–
Erectile dysfunction (n=13)	11 (84.6%)	1 (7.7%)	1 (7.7%)	–
Disorders of ejaculation (n=14)	13 (92.9%)	1 (7.1%)	–	–
Low sexual desire (n=14)	12 (85.6%)	2 (14.4%)	–	–
Penile curvature (n=14)	13 (92.9%)	1 (7.1%)	–	–
Penile size abnormalities (n=14)	12 (85.6%)	1 (7.2%)	1 (7.2%)	–
Priapism (n=13)	11 (84.6%)	2 (15.4%)	–	–
Male infertility (n=14)	12 (85.6%)	1 (7.2%)	1 (7.2%)	–
EAU guideline recommendations (n=80)	80 (86.3%)	8 (10%)	3 (3.8%)	–
Male hypogonadism (n=22)	18 (81.8%)	2 (9.1%)	2 (9.1%)	–
Erectile dysfunction (n=8)	7 (87.5%)	1 (12.5%)	–	–
Disorders of ejaculation (n=6)	6 (100%)	–	–	–
Penile curvature (n=15)	12 (80.0%)	2 (13.3%)	1 (6.7%)	–
Penile size abnormalities (n=10)	9 (90.0%)	1 (10.0%)	–	–
Priapism (n=8)	7 (87.5%)	1 (12.5%)	–	–
Male infertility (n=11)	10 (90.9%)	1 (9.1%)	–	–

4 points: completely correct, 3 points: correct but insufficient, 2 points: misleading information as well as correct information, 1 point: completely incorrect

EAU: European Association of Urology

**Supplementary Table 1.** Frequently Asked Questions Related Andrology

**Frequently Asked Questions**
1- What is male hypogonadism?
2- What are the primary causes of male hypogonadism?
3- What are the common symptoms of male hypogonadism?
4- How does age impact the likelihood of developing hypogonadism?
5- Can male hypogonadism affect fertility?
6- How is male hypogonadism diagnosed?
7- Is testosterone replacement therapy (TRT) an effective treatment?
8- What are the potential benefits of TRT for male hypogonadism?
9- Are there any risks or side effects associated with TRT?
10- Can lifestyle changes help manage male hypogonadism?
11- Is it possible to reverse male hypogonadism with treatment?
12- How does male hypogonadism impact overall health?
13- What is erectile dysfunction (ED)?
14- What causes erectile dysfunction?
15- How common is ED?
16- What are the risk factors for developing ED?
17- Can psychological factors contribute to ED?
18- How is ED diagnosed?
19- What treatment options are available for ED?
20- Is medication effective in treating ED?
21- Can lifestyle changes improve erectile function?
22- What role does communication play in managing ED?
23- Are there surgical options for severe cases of ED?
24- Can ED be a sign of other underlying health conditions?
25- How does age impact the likelihood of ED?
26- What are disorders of ejaculation?
27- What causes delayed ejaculation?
28- What is premature ejaculation?
29- How are disorders of ejaculation diagnosed?
30- Can psychological factors contribute to these disorders?
31- What are the treatment options for delayed ejaculation?
32- How is premature ejaculation managed?
33- Are there medications specifically designed for these disorders?
34- Can therapy or counseling be effective in treating these disorders?
35- Can relationship issues impact ejaculation disorders?
36- Is it possible for these disorders to resolve on their own?
37- Are there exercises or techniques to help improve ejaculation control?
38- How do medical conditions affect disorders of ejaculation?
39- Can lifestyle changes play a role in managing these disorders?
40- What is low sexual desire in males?
41- What is Male Hypoactive Sexual Desire Disorder (HSDD)?
42- What factors can contribute to low sexual desire?
43- Is low sexual desire a common issue?
44- How is Male HSDD diagnosed?
45- Can hormonal imbalances lead to low sexual desire?
46- What role do psychological factors play in Male HSDD?
47- Are there medications or therapies for Male HSDD?
48- Can communication and relationship issues affect sexual desire?
49- How does age impact male sexual desire?
50- Are there natural remedies that can boost sexual desire?
51- Can lifestyle changes improve low sexual desire?
52- Is low sexual desire reversible?
53- Can excessive stress and anxiety cause Male HSDD?
54- What causes penile curvature?
55- Is a certain amount of curvature normal?
56- When does penile curvature become a medical concern?
57- How is penile curvature evaluated and diagnosed?
58- Can Peyronie's disease cause penile curvature?
59- What are the treatment options for penile curvature?
60- Can penile curvature affect sexual function?
61- Are there surgical interventions for severe cases of penile curvature?
62- Can lifestyle changes prevent or manage penile curvature?
63- Can penile curvature impact self-esteem and body image?
64- Is penile curvature more common with age?
65- How does injury or trauma contribute to penile curvature?
66- Can certain sexual practices cause penile curvature?
67- How do medical conditions affect penile curvature?
68- What is dysmorphophobia in relation to penile size?
69- How do individuals perceive penile size abnormalities?
70- What factors contribute to concerns about penile size?
71- Is there a "normal" range for penile size?
72- How does dysmorphophobia impact psychological well-being?
73- Can media and societal influences contribute to dysmorphophobia?
74- Are there medical treatments to address penile size abnormalities?
75- How can therapy or counseling help individuals with dysmorphophobia?
76- Can penile size abnormalities affect sexual function?
77- Is surgery an option for those seeking to change penile size?
78- Are there safe methods to increase penile size?
79- Can exercises or devices help improve penile size?
80- Is dysmorphophobia specific to penile size or part of body dysmorphic disorder?
81- How can healthcare professionals address concerns about penile size?
82- What is priapism?
83- What causes priapism?
84- How is priapism different from a normal erection?
85- Are there different types of priapism?
86- Can certain medications trigger priapism?
87- Is priapism a medical emergency?
88- How is priapism diagnosed?
89- What are the potential complications of untreated priapism?
90- What treatment options are available for priapism?
91- Can draining blood from the penis resolve priapism?
92- Can lifestyle changes prevent recurrent episodes of priapism?
93- Can psychological factors contribute to priapism?
94- Is priapism more common in certain age groups?
95- What is male infertility?
96- What causes male infertility?
97- How common is male infertility?
98- Can medical conditions or treatments contribute to infertility?
99- What are the risk factors for male infertility?
100- How is male infertility diagnosed?
101- Can lifestyle factors impact male fertility?
102- What treatment options are available for male infertility?
103- Can assisted reproductive techniques help overcome male infertility?
104- Is there a link between male infertility and sexual dysfunction?
105- Can hormonal imbalances affect male fertility?
106- Does age play a role in male infertility?
107- How does diet and nutrition impact male fertility?
108- Can environmental factors contribute to male infertility?

Male hypogonadism (Between 1^st^ -12^th^ questions), Erectile dysfunction (Between 13^th^ – 25^th^ questions), Disorders of ejaculation (Between 26^th^ -39^th^ Questions), Low sexual desire and male hypoactive sexual desire disorder (Between 40^th^ -53^th^ Questions), Penile curvature (Between 54^th^ – 67^th^ Questions), Penile size abnormalities and dysmorphophobia (Between 68^th^ – 81^th^ Questions), Priapism (Between 82^th^ – 94^th^ Questions), Male infertility (Between 95^th^ – 108^th^ Questions)

**Supplementary Table 2.** Questions Related to Guideline Recommendations by the European Association of Urology (EAU)

**European Association of Urology (EAU) Guideline Questions**
1- Which factors should be examined to determine if they can interfere with testosterone production or action?
2- When should total testosterone levels be assessed, and under what conditions, to ensure accuracy?
3- Is it recommended to conduct total testosterone retesting ?
4- What is the established threshold for diagnosing late-onset hypogonadism (LOH) using total testosterone?
5- Is there a meaningful benefit in taking into account sex hormone-binding globulin and free testosterone?
6- Which test is utilized to distinguish between primary and secondary hypogonadism?
7- If there's a presence of low sexual desire (or other indicative signs/symptoms) along with low or low-normal testosterone, what measurement should be taken into consideration?
8- What procedure is recommended for individuals with secondary hypogonadism, elevated PRL levels, specific symptoms suggesting a pituitary mass, and/or other anterior pituitary hormone deficiencies?
9- In which patient group should screening for late-onset hypogonadism (LOH), including in individuals with type 2 diabetes (T2DM), be conducted?
10- Should structured interviews and self-reported questionnaires be used for systematic screening for LOH, considering their low specificity?
11- What therapy should not be used in eugonadal men?
12- Which therapy should be used as the first-line treatment in patients with symptomatic hypogonadism and mild erectile dysfunction (ED)?
13- Which therapy should be used for severe depressive symptoms and osteoporosis?
14- Is it appropriate to use testosterone therapy to enhance cognition, vitality, and physical strength in aging men?
15- What steps should be taken for addressing organic causes of hypogonadism?
16- How should patients be approached regarding treatment options for hypogonadism?
17- What guidance should be provided to patients concerning the utilization of testosterone therapy in men who have undergone treatment for breast cancer?
18- What should be assessed before initiating testosterone therapy?
19- What should be excluded or assessed for venous thromboembolism before initiating testosterone therapy?
20- What should be monitored after the initiation of testosterone therapy, and how often should this monitoring take place thereafter?
21- What actions should be taken if the hematocrit level exceeds 54% during testosterone therapy?
22- What is the recommended timeframe for evaluating patients with polycythemia vera and those at a higher risk of developing elevated hematocrit levels?
23- What actions or steps should be taken for a patient presenting with erectile dysfunction (ED)?
24- What assessment tool or approach should be used in relation to erectile dysfunction (ED) to evaluate all sexual function domains and the impact of a particular treatment method?
25- What steps should be taken during the initial assessment of men with erectile dysfunction (ED) to identify underlying medical conditions and comorbid genital disorders that could be linked to ED?
26- What psychological approach, including involving the partner, should be utilized in combination with medical treatment to optimize treatment outcomes?
27- What are the recommended steps to be taken for the treatment of erectile dysfunction?
28- How should the diagnosis and classification of premature ejaculation (PE) be conducted?
29- Should routine laboratory or neurophysiological tests be conducted for erectile dysfunction (ED)?
30- Which condition should take priority for treatment: premature ejaculation or erectile dysfunction (ED)?
31- Which condition should be addressed first in terms of treatment: premature ejaculation or genitourinary infection?
32- What are the recommended first-line treatments for lifelong premature ejaculation (PE)?
33- Is it advisable to use off-label tramadol with caution as a potential on-demand alternative to on-demand SSRIs for the treatment of certain conditions?
34- Should the use of certain therapies be considered, either alone or in combination with other treatments, for patients with premature ejaculation (PE) who do not have erectile dysfunction (ED)?
35- What are the recommended management recommendations for dealing with recurrent hematospermia?
36- What are the recommended treatments for addressing low sexual desire?
37- What aspects should be examined in the medical and sexual history of patients with Peyronie's disease (PD)?
38- What specific aspects should be examined during the physical examination of patients with Peyronie's disease (PD)?
39- Under what circumstances should conservative treatment be offered to patients with Peyronie's disease?
40- What information should patients be provided with regarding all available treatment options and potential outcomes before initiating any form of treatment?
41- What information should patients be provided with regarding all available treatment options and potential outcomes before initiating any form of treatment?
42- Is it considered appropriate to use vitamin E, potassium para-aminobenzoate (potaba), tamoxifen, pentoxifylline, colchicine, and acetyl esters of carnitine for treating Peyronie’s disease (PD)?
43- What should be administered to treat penile pain during the acute phase of Peyronie's disease (PD)?
44- What options should be offered to patients with stable dorsal or lateral curvature greater than 30 degrees who are seeking a minimally invasive procedure?
45- Is it advised to provide intralesional treatment with steroids to reduce penile curvature, plaque size, or pain?
46- Is it recommended to offer Extracorporeal Shockwave Therapy (ESWT) to improve penile curvature and reduce plaque size?
47- When should surgery be performed for Peyronie's disease (PD)?
48- What aspects should be assessed prior to surgery for Peyronie's disease (PD)?
49- Should the sliding techniques be used for peyronie's disease?
50- Is it recommended to use synthetic grafts in Peyronie's disease (PD) reconstructive surgery?
51- What should be used, with or without any additional straightening procedures, in Peyronie's disease (PD) patients with erectile dysfunction (ED) who do not respond to pharmacotherapy?
52- What aspects should be assessed in patients with a normal-sized penis who are complaining of short penile size?
53- What course of action should be taken for patients with suspected Body Dysmorphic Disorder (BDD) in terms of mental health counseling?
54- In which cases should psychotherapy be considered as part of the approach to penile augmentation?
55- What treatment option should be utilized to restore penile size in boys with micropenis or disorders of sex development?
56- Is it considered appropriate to use testosterone therapy or other hormonal therapies to increase penile size in men after puberty?
57- What are the recommended approaches for dealing with Adult Acquired Buried Penis (AABP)?
58- What steps should be taken to address congenital intrinsic penile shortness?
59- Is it considered appropriate to offer penile prosthesis implantation, penile disassembly, or sliding technique to patients who are seeking penile lengthening options?
60- Is it considered safe to use silicone, paraffin, and petroleum jelly (Vaseline) for penile girth enhancement?
61- Is it considered acceptable to use grafts for penile girth enhancement?
62- Which method or factor can aid in determining the subtype of priapism?
63- What are the recommended laboratory testing for diagnosing ischemic priapism?
64- What procedure or action should be performed when planning embolization for the management of non-ischemic priapism?
65- How should the management of ischemic priapism be initiated?
66- In cases of priapism secondary to intracavernous injections of vasoactive agents, what is the initial step that should be taken?
67- Should ischaemic priapism associated with sickle cell disease be treated in the same manner as other cases of ischaemic priapism?
68- If a shunt procedure has been performed, is it recommended to delay the implantation of a penile prosthesis?
69- What are the recommended treatment for stuttering priapism?
70- What are the essential components of a male infertility evaluation?
71- In which condition should standard karyotype analysis and genetic counseling be considered for diagnostic purposes in semen analysis?
72- Is it useful to test for Y-chromosome microdeletions in men with pure obstructive azoospermia?
73- When might Y-chromosome microdeletion testing be offered to men?
74- What should be attempted in patients with complete deletions that include the aZFa and aZFb regions?
75- In men with structural abnormalities of the vas deferens (unilateral or bilateral absence without renal agenesis), what should both the man and his partner be tested for?
76- What should be performed in the assessment of couples experiencing recurrent pregnancy loss from natural conception and assisted reproductive technology (ART), as well as in men with unexplained infertility?
77- What should be performed if there is suspicion of a partial or complete distal obstruction?
78- Should imaging be considered for detecting renal abnormalities in men who have structural abnormalities of the vas deferens and no evidence of cystic fibrosis transmembrane conductance regulator abnormalities?
79- How should hypogonadotropic hypogonadism (secondary hypogonadism), including congenital causes, be treated?
80- Which factors should be examined to determine if they can interfere with testosterone production or action?
